# Anti‐VEGF‐A/ANG2 combotherapy limits pathological angiogenesis in the eye: a replication study

**DOI:** 10.15252/emmm.201910362

**Published:** 2019-04-30

**Authors:** Anne Wolf, Thomas Langmann

**Affiliations:** ^1^ Laboratory for Experimental Immunology of the Eye Department of Ophthalmology Faculty of Medicine and University Hospital Cologne University of Cologne Cologne Germany; ^2^ Center for Molecular Medicine Cologne (CMMC) Cologne Germany

**Keywords:** Neuroscience, Pharmacology & Drug Discovery, Vascular Biology & Angiogenesis

## Abstract

Neovascular and inflammatory retinal diseases including wet age‐related macular degeneration (AMD) can cause severe vision loss among the elderly. Simultaneous neutralization of vascular endothelial growth factor‐A (VEGF‐A) and angiopoietin 2 (ANG2) is envisioned as a novel candidate approach to treat wet AMD with better efficacy. However, earlier published data from a genetic mouse model showed data aberrations (Regula *et al*, 2016). In this issue of *EMBO Molecular Medicine*, Foxton *et al* (2019) have provided compelling evidence replicating the data and confirming the overall concept that VEGF‐A/ANG2 combotherapy is effective in suppressing retinal neovascularization.

Choroidal neovascularization is a sight‐threatening late‐stage complication of age‐related macular degeneration, a prevalent multifactorial eye disease of the elderly (Wong *et al*, 2014). Anti‐vascular endothelial growth factor biologics are now the standard of care for wet AMD and other ocular neovascular diseases including diabetic macular edema, retinal vein occlusion, and retinopathy of prematurity (Kim & D'Amore, 2012). These therapies have significantly contributed to preserve vision in many patients. However, a subgroup of patients loses initial vision gains due to recurrence of neovascular leakage (Comparison of Age‐related Macular Degeneration Treatments Trials Research Group *et al*, 2016). Moreover, the high frequency of intraocular injections and potential side effects of chronic VEGF neutralization may limit the efficacy (Nguyen *et al*, 2018). Another aspect currently not addressed by established anti‐VEGF therapies is the chronic retinal immune response (Akhtar‐Schafer *et al*, 2018). Therefore, there is a high need to identify additional factors that regulate retinal neovascularization.

The cytokine ANG2 is a perfect therapeutic candidate for wet AMD as it functions in both angiogenesis and immune activation (Scholz *et al*, 2015), two processes that are involved in ocular pathological neovascularization. In the mouse retina, Ang2 is required for early vascular development and Ang2‐deficient animals lack ischemia‐induced neovascularization (Hackett *et al*, 2002). In human eyes, higher levels of ANG2 correlate with disease severity in wet AMD (Ng *et al*, 2017). Increased intraocular ANG2 levels were also detected in patients with diabetic retinopathy and retinal vein occlusion, indicating a potential medical significance of targeting ocular ANG2 (Regula *et al*, 2016).

In the same study, Regula *et al* reported preclinical data from a mouse model of aberrant retinal angiogenesis, where combined inhibition of VEGF‐A/ANG2 with a bispecific antibody strongly reduced vascular leakage, immune reactivity, and apoptosis. Intravitreal injections of a related human monoclonal antibody (RG7716) were also effective in a laser‐induced CNV model in non‐human primates. In the aftermath of the publication and with the help of expert reviewers, it came to the attention of *EMBO Mol Med* editors that two different microscopic images showed overlapping staining signals and insufficiently labeled bar charts that questioned the validity of the mouse experiments. *EMBO Mol Med* published an expression of concern stating that these data require further analysis and verification (Regula *et al*, 2017). As a response to the transparent editorial process and rigorous peer review at *EMBO*, an independent research team at Roche Pharma Research and Early Development (pRED) replicated the concerned mouse experiments (a subset of the authors of the Regula *et al* study had affiliations to Roche).

In Basel, the Roche pRED scientists established a new colony of JR5558 animals, a mutant mouse strain with spontaneous CNV that was supplied from Charles River, Germany. Then, blinded data analyses of fluorescein angiography to determine vessel leakage and TUNEL stains to detect cell death were performed with 9–10 animals per group, compared to six animals per group in the initial Regula *et al* study. Therefore, these validation experiments had sufficiently high statistical power as can be seen from the bar graphs showing individual data points. A comparison of the sham group with single and combined antibody treatments finally showed a superior efficacy of simultaneous VEGF‐A/ANG2 inhibition in suppressing choroidal neovascularization (Foxton *et al*, 2019).

The successful replication and scientific validity of preclinical data are of enormous importance for future clinical trials in this challenging field of neovascular retinal diseases. Here, successful phase 1 (NCT01941082 at www.clincaltrials.gov) and phase 2 (NCT02484690) proof‐of‐concept trials were performed with the anti‐VEGF‐A/ANG2 bispecific antibody (RG7716 alias RO6867461). The efficacy of this molecule now termed faricimab will be compared with the well‐established VEGF/PGF trap Aflibercept/Eylea in larger phase 3 trials (NCT03823300 and NCT03823287). We hope for the patients that the repertoire of effective biologics will be expanded and the clinical outcomes will be significantly improved in the near future.

## Conflict of interest

TL serves in advisory boards for Bayer, Novartis, and Roche that develop drugs for retinal degenerative and neovascular diseases.
